# Parallel Faster-X Evolution of Gene Expression and Protein Sequences in *Drosophila*: Beyond Differences in Expression Properties and Protein Interactions

**DOI:** 10.1371/journal.pone.0116829

**Published:** 2015-03-19

**Authors:** Ana Llopart

**Affiliations:** 1 Department of Biology, The University of Iowa, Iowa City, Iowa, United States of America; 2 Interdisciplinary Graduate Program in Genetics, The University of Iowa, Iowa City, Iowa, United States of America; North Carolina State University, UNITED STATES

## Abstract

Population genetics models predict that the *X* (or *Z*) chromosome will evolve at faster rates than the autosomes in *XY* (or *ZW*) systems. Studies of molecular evolution using large datasets in multiple species have provided evidence supporting this faster-X effect in protein-coding sequences and, more recently, in transcriptomes. However, X-linked and autosomal genes differ significantly in important properties besides hemizygosity in males, including gene expression levels, tissue specificity in gene expression, and the number of interactions in which they are involved (i.e., protein-protein or DNA-protein interactions). Most important, these properties are known to correlate with rates of evolution, which raises the question of whether differences between the *X* chromosome and autosomes in gene properties, rather than hemizygosity, are sufficient to explain faster-X evolution. Here I investigate this possibility using whole genome sequences and transcriptomes of *Drosophila yakuba* and *D. santomea* and show that this is not the case. Additional factors are needed to account for faster-X evolution of both gene expression and protein-coding sequences beyond differences in gene properties, likely a higher incidence of positive selection in combination with the accumulation of weakly deleterious mutations.

## Introduction

Population genetics theory predicts that under certain conditions the *X* chromosome will evolve at faster rates than the autosomes in *XY* (or *ZW*) systems, an effect known as faster-X [1,2]. In a population of diploids, newly arisen autosomal mutations are mostly found in heterozygotes and, if recessive for fitness, their effects are masked by the ancestral variants. In contrast, if these mutations arise on the *X* chromosome and are beneficial, natural selection can drive them to fixation more efficiently because their fitness effects will be fully exposed in hemizygous males. As a result, the *X* chromosome is expected to accumulate adaptive mutations at a faster rate than the autosomes. This difference is expected to be more extreme for mutations that affect only males [3].

Yet in *XY* (or *ZW*) systems faster-X evolution can also result from new mutations that are weakly deleterious [1,2,4,5]. If a population contains equal numbers of breeding females and males, the effective population size of the *X* chromosome (*N*_*eX*_) is expected to be ¾ the effective population size of the autosomes (*N*_*eA*_) (all else being equal). This reduced *N*_*eX*_ results in increased genetic drift and, as a consequence, weakly deleterious mutations that are partially dominant attain higher frequencies in the population and reach fixation more often when X-linked than when autosomal, thus producing faster-X. Note that strongly deleterious mutations are eliminated from the population by natural selection before they can reach fixation regardless of whether they are X-linked or autosomal.

The likelihood of faster-X being driven by either beneficial or weakly deleterious mutations is strongly influenced by the *N*_*eX*_ to *N*_*eA*_ ratio [4,5]. Taxa with small *N*_*eX*_/*N*_*eA*_, such as several species of birds, are expected to be more susceptible to faster-X associated with the fixation of weakly deleterious mutations [6], although positive selection has also been invoked [7,8]. Cases where faster-X is due to weakly deleterious mutations should be also accompanied by a reduction of neutral polymorphism on the *X* chromosome due to the smaller *N*_*eX*_. On the other hand, the observation of faster-X in species with *N*_*eX*_/*N*_*eA*_ close to, or slightly above, 1 is more easily explained by a higher incidence of adaptive evolution on the *X* chromosome [4,5]. The *N*_*eX*_ to *N*_*eA*_ ratio is influenced by multiple factors besides sex ratios, including mating system, differences between the two sexes in the strength of sexual selection, or disparities in recombination rates and linkage effects between the *X* chromosome and autosomes [4,9–13].

Regardless of the evolutionary forces underlying faster-X (selection on beneficial or weakly deleterious mutations), the evidence for this effect was initially controversial. In *Drosophila* early attempts to evaluate the faster-X evolution of protein-coding sequences produced inconsistent results [14–19] but more recent studies did find clear statistical support [20–31]. Importantly the strongest evidence for faster-X is observed in male-biased genes (i.e., genes expressed at higher levels in males than in females, MBGs) [20,26,29]. To date, accelerated rates of evolution for X-linked proteins have been documented in a wide range of taxa, including primates [32–36], mice [37,38], birds [6–8], snakes [39], worms [40], silkmoths [41] and aphids [42], which are an X0 system. Faster-X evolution of gene expression has been shown in primates [35] and more recently in *Drosophila* [43–45].

One limitation of all studies of faster-X is that X-linked and autosomal genes may not be directly comparable [46,47]. MBGs have been proposed to be underrepresented on the *Drosophila X* chromosome [48–50]. Recent studies show that the lack of dosage compensation in the *Drosophila* male germline is an important factor explaining the apparent deficit of X-linked MBGs [51,52]. Levels of gene expression and tissue specificity are significantly different in X-linked than in autosomal genes of *D. melanogaster* [47,52–56]. Transcriptional networks also show unique characteristics for genes on the *X* chromosome [57,58]. Most important, variation in these properties correlates significantly with rates of evolution [46,59–65].

In a comprehensive study of the 12 *Drosophila* genomes, Larracuente *et al*. (2008) showed that gene expression levels were negatively correlated with evolutionary rate, and that tissue specificity in gene expression was independently positively correlated with the rate of evolution [59]. Protein interactions and protein divergence also showed a negative association in *Drosophila* and yeast [59,64,65]. These observations raise the question of whether differences between X-linked and autosomal genes in expression properties (i.e., levels and tissue specificity) and protein interactions, rather than hemizygosity, could entirely explain faster-X. To evaluate this possibility, I examined genomewide gene expression divergence between *Drosophila yakuba* and *D. santomea* and whole-genome patterns of protein-coding sequence divergence based on a newly obtained *D. santomea* genome sequence. *D. santomea* started diverging allopatrically from its common ancestor with *D. yakuba* ~400,000 years ago [66,67] on the African island of São Tomé where today the two species form a hybrid zone [68]. I show that gene expression properties are good predictors of both gene expression and protein-coding sequence divergence between species, but they are not sufficient to explain faster-X for MBGs. Additional evolutionary factors are thus necessary to account for the rapid evolution of MBGs on the *X* chromosome in this system.

## Materials and Methods

### Generating a *D. santomea* genome sequence

To generate a high quality, high coverage sequence of the *D. santomea* genome, I obtained Illumina reads from 5 different strains (STO.4, STO.18, QUIJA 650.1, COST1235.1 and CAR1600.3; details on the strains are reported in [67]) according to the manufacturer guidelines (“Preparing Samples for Sequencing Genomic DNA”, Part # 1003806 Rev. B, Illumina, San Diego, CA). High molecular weight DNA was isolated from 30 flies following protocol 47 in Ashburner (1989) with minor modifications [69]. Cluster generation and sequencing (75 to 101-bp single-end reads) were carried out in Illumina GAII/HiSeq2000 instruments at the Iowa State DNA Facility (Iowa State University, Ames, IA). Trimming and filtering were done using SAMTools v1.4 [70] and custom scripts.

To obtain a *D. santomea* syntenic sequence, 208.1 million filtered reads were aligned to the *D. yakuba* genome project sequence (dyak_r1.3_FB2011_08; http://flybase.org/) [71] using BWA [72]. SAMTools was used to generate pileup and fasta files for each chromosome arm from BAM files. Only nucleotides with coverage greater than 7× and phred-scaled base consensus (or variant) quality greater than *Q* = 40 were included in the final *D. santomea* synthetic and syntenic sequence. The average read depth of this high quality sequence for *D. santomea* is 145.9× with an average phred-scaled quality of *Q* = 242, covering 91.8% of the *D. yakuba* reference genome [71]. To extract the *D. santomea* coding sequences of all genes, I used Galaxy [73–75] and the exon-intron annotation of *D. yakuba* (dyak_r1.3_FB2011_08; http://flybase.org/). All *D. santomea* reads have been deposited in the NCBI Short Read Archive (SRA) BioProject SRP049565.

### Evolutionary Analysis

To estimate gene expression levels, I used data on transcript abundance from Llopart (2012) [43]. Briefly, gene expression was measured in 1-day old *D. yakuba* and *D. santomea* adult males using whole-genome, custom-designed *D. yakuba* arrays (11,530 genes analyzed; 60-mer probes; 10 probes/gene). For any given gene, expression level was calculated as the average signal (in Log_2_) of the two species. Gene expression divergence between *D. yakuba* and *D. santomea* and gene expression polymorphism were obtained from [43]. To gauge tissue specificity in gene expression, I calculated the tissue specificity index (*τ*), which takes into account the number of tissues in which a gene is expressed and the relative expression levels in each tissue [76]. Genes expressed in many different tissues tend to show low *τ* values, and genes expressed in a narrow number of tissues, high *τ* values. Information on gene expression for different tissues was inferred from the analysis of all 25 tissues included in *D. melanogaster* FlyAtlas (http://www.flyatlas.org/) [77]. These tissues are brain, head, eye, thoracicoabdominal ganglion, salivary gland, crop, midgut, tubule, hindgut, heart, fat body, ovary, testis, male accessory glands, virgin spermatheca, mated spermatheca, adult carcass, larval CNS, larval salivary gland, larval midgut, larval tubule, larval hindgut, larval fat body, larval trachea and larval carcass. To determine the number of physical interactions, I used information derived from the strict gene interaction network of *D. melanogaster* proposed by Hansen and Kulathinal (2013) [58]. This is an empirically driven network of physical interactions based on data derived from six experiments, including yeast-two-hybrid and transcription-factor CHIP-seq analyses from the modENCODE project; the network contains 12,749 genes and 486,138 interactions [58].

To estimate protein-coding sequence divergence, I calculated the number of nonsynonymous (*d*_*N*_) and synonymous (*d*_*S*_) substitutions per site between *D. yakuba* and *D. santomea* using the codeml program implemented in PAML v4.5 [78,79]. The equilibrium codon frequencies were calculated from average nucleotide frequencies at third codon positions (codon substitution model F3×4). A single *d*_*N*_/*d*_*S*_ ratio (ω) was assumed for all lineages and across sites (Model 0). There were 16,082 annotated genes in the *D. yakuba* reference sequence (dyak_r1.3_FB2011_08; http://flybase.org/) [71] and, after excluding genes located on either heterochromatic regions or in unknown chromosomal locations, I applied codeml to 14,682 genes. In 316 genes I found stop codons in the *D. santomea* sequence, which are likely due to either inaccuracy in the current annotation of *D. yakuba*, sequencing errors, or differences between species in protein length or exon-intron structure. There were three *D. yakuba* genes with no orthologous sequences recovered in *D. santomea*. Finally I also excluded from the analysis 350 genes with 0 synonymous changes between *D. yakuba* and *D. santomea* and 354 genes with ω ≥ 9, as they tend to generate unreliable estimates of divergence. I obtained *d*_*N*_, *d*_*S*_ and ω in two sets of genes. The first set contains all 13,659 genes sequenced in both species, without stop codons in *D. yakuba* and *D. santomea*, and with reliable estimates of divergence. The second set of genes analyzed contains the 9,203 out of the 13,659 genes for which there was information on gene expression level [43], tissue specificity in gene expression, and the number of protein interactions.

## Results and Discussion

### Faster-X evolution of male-biased gene expression

A previous study reported faster-X evolution of male-biased gene expression in *D. yakuba* and *D. santomea* [43]. The same significant trend is detected in the current dataset of 9,697 genes with known gene expression levels, tissue specificity, and the number of protein interactions (1,537 X-linked and 8,160 autosomal) (Mann-Whitney *U* test, *P* = 2.5×10^-8^; see S1 Fig). To determine whether these properties are significantly associated with gene expression divergence in *D. yakuba* and *D. santomea*, I examined their distributions across the genome. As expected, expression levels and the number of protein interactions are negatively correlated with gene expression divergence (Spearman’s rank correlation *ρ* = -0.25 for levels and *ρ* = -0.19 for protein interactions, both *P* < 1×10^-6^) while tissue specificity shows a positive correlation (Spearman’s rank correlation *ρ* = 0.23, *P* < 1×10^-6^). Similar associations have been reported in other *Drosophila* species [59 and references therein,^64^,^65^]. Most important, X-linked and autosomal genes differ in these properties.

**Fig 1:**
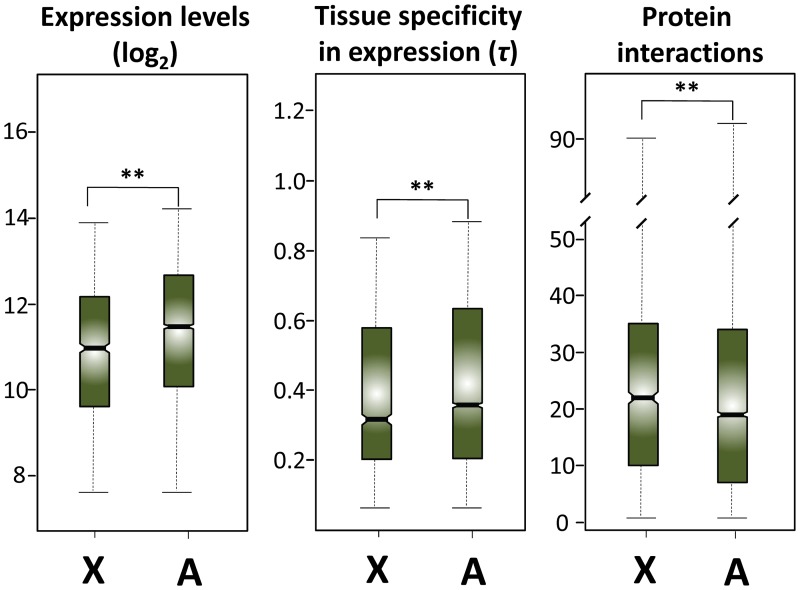
Comparisons of gene expression levels, tissue specificity in gene expression, and number of protein interactions between X-linked (*X*) and autosomal (*A*) genes. The horizontal line inside each box indicates the median. The length of the box and the whiskers represent 50% and 90% confidence intervals, respectively. Asterisks indicate statistically significant differences (Mann-Whitney *U* test; *P* < 1×10^-12^ for expression levels, *P* = 1.6×10^-4^ for tissue specificity, and *P* = 3×10^-4^ for protein interactions).

X-linked genes are expressed at significantly lower levels in adult males, show less tissue specificity (i.e., expressed in more tissues), and are involved in more protein interactions than autosomal genes (Fig. 1). Based on these differences and genomewide correlations, tissue specificity and protein interactions may be in fact masking faster-X in this dataset. In contrast, lower expression in X-linked than in autosomal genes could potentially explain faster-X evolution. Altogether these results underline the need to control for differences between the *X* chromosome and autosomes in expression properties (i.e., levels and tissue specificity) and protein interactions when assessing faster-X.

To evaluate whether the rapid evolution of X-linked male-biased gene expression is detectable after controlling for differences between the *X* chromosome and autosomes in levels of gene expression, tissue specificity and the number of protein interactions, I performed multiple-regression analysis (Table 1). Residuals of gene expression divergence are significantly greater for X-linked than for autosomal MBGs; a pattern not detected in nonsex-biased genes (NBGs) suggesting a faster-X effect beyond differences in gene properties (Mann-Whitney *U* test, *P* = 0.002 for MBGs and Mann-Whitney *U* test, *P* = 0.08 for NBGs). In addition, I carried out an analysis that is free of the assumptions associated with standard linear regression models and enables comparisons of gene expression divergence between subsets of genes with similar expression properties and number of protein interactions. Expression levels, tissue specificity, and the number of protein interactions were each subdivided into 10 equal-size categories and all MBGs were assigned to their corresponding combined (3-dimensions) category. For each category, I then obtained mean expression divergence for X-linked and autosomal MBGs separately. Finally, I applied a nonparametric test to determine whether there was an overrepresentation of categories in which X-linked MBGs show higher gene expression divergence than autosomal MBGs. (Only categories that contained both X-linked and autosomal MBGs were considered.) This analysis shows that X-linked MBGs have consistently higher gene expression divergence than autosomal MBGs (Wilcoxon matched pairs test, *T* = 4772.0, *P* = 0.0032) when sharing expression properties and number of protein interactions. Results from both types of analyses imply that the observed excess of gene expression divergence in MBGs on the *X* chromosome remains significant after taking into account differences among genes in properties. Note that while estimates of expression levels are based directly on transcript abundance in *D. yakuba* and *D. santomea*, tissue specificity and protein interactions are inferred from the close relative *D. melanogaster*. However potential errors in this inference could not explain our results, as they are not expected to impact differentially X-linked and autosomal genes.

**Table 1:** Parameters of multiple-regression models and partial correlations for gene expression divergence.

	*R* (*P*)	b*Lev (*P*)	b*Spe (*P*)	b*Int (*P*)
MBGs	0.33 (<1×10^-12^)	-0.30 (<1×10^-12^)	0.07 (0.003)	-0.04 (0.043)
NBGs	0.35 (<1×10^-12^)	-0.25 (<1×10^-12^)	0.17 (<1×10^-12^)	-0.03 (0.032)
All	0.39 (<1×10^-12^)	-0.28 (<1×10^-12^)	0.19 (<1×10^-12^)	-0.027 (0.004)

*Note*: *R*, correlation coefficient; *P*, Probability; Lev, average gene expression levels; Spe, tissue specificity in gene expression estimated according to Yanai *et al*. (2005) [76]; Int, protein interactions identified from Hansen and Kulathinal (2012) [58]; MBGs, male-biased genes; NBGs, nonsex-biased genes; All, all genes.

**Fig 2:**
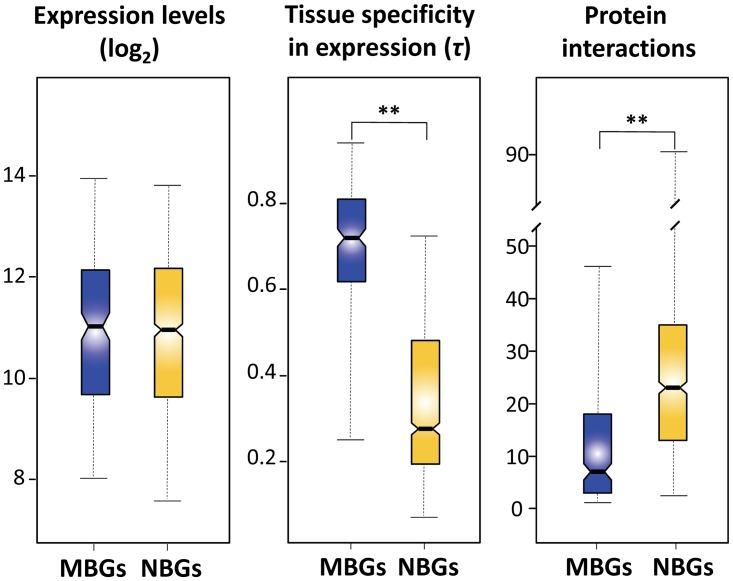
Comparisons of gene expression levels, tissue specificity in gene expression, and number of protein interactions between X-linked MBGs (blue) and X-linked NBGs (yellow). Asterisks indicate statistically significant differences (Mann-Whitney *U* test; *P* < 1×10^-32^ for tissue specificity and *P* = 7.3×10^-29^ for protein interactions). (See Fig. 1 legend for explanation of box plots.)

**Fig 3:**
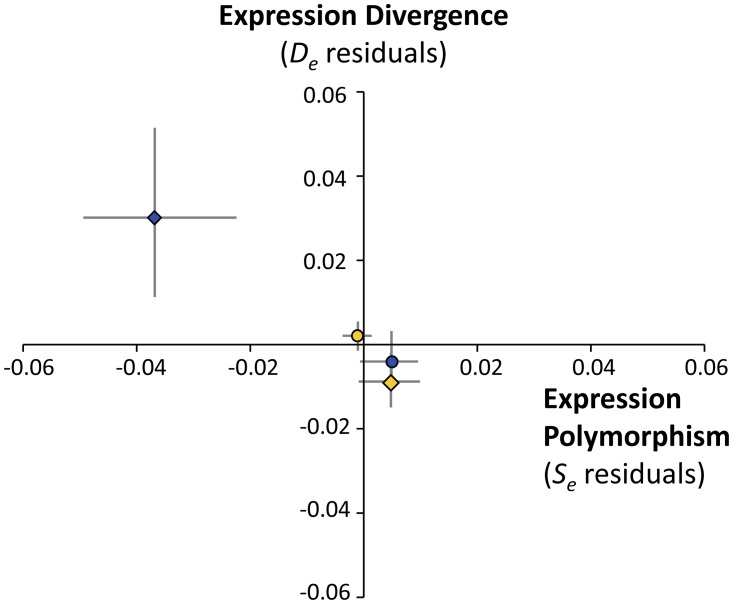
Multiple-regression residuals of gene expression divergence and polymorphism (mean) for X-linked male-biased genes (MBGs) (blue diamonds), autosomal MBGs (blue circles), X-linked nonsex-biased genes (NBGs) (yellow diamonds), and autosomal NBGs (yellow circles). Grey lines represent 90% confidence limits of the residual means.

The excess of gene expression divergence for X-linked MBGs could be the result of either positive selection or increased genetic drift associated with *N*_*eX*_ < *N*_*eA*_ and a higher rate of accumulation of weakly deleterious mutations. The analysis of silent nucleotide polymorphism in 26 nuclear regions sequenced in *D. yakuba* and *D. santomea* indicated that the *N*_*eX*_ to *N*_*eA*_ ratio is ~0.63 [67], which opens the possibility that faster-X evolution of gene expression could be the result of increased genetic drift on the *X* chromosome. To test this possibility, I investigated gene expression polymorphism in both MBGs and NBGs on the *X* chromosome [43]. X-linked NBGs are more polymorphic than X-linked MBGs (median gene expression polymorphism is 0.21 for NBGs and 0.16 for MBGs; Mann-Whitney *U* test, *P* = 0.0002), even though the latter show increased tissue specificity and are involved in fewer protein interactions (Fig. 2). Indeed this difference remains significant after correcting for variation in expression properties and protein interactions on the *X* chromosome using multiple-regression analysis (Mann-Whitney *U* test, *P* = 7.3×10^-6^ in analysis of residuals; Table 2). Although the precise relationship between nucleotide changes and variation in gene expression is not yet fully understood, the contrasting patterns of gene expression divergence and polymorphism in MBGs and NBGs suggest that a fraction of beneficial mutations contributes to faster-X evolution of male-biased gene expression in *D. yakuba* and *D. santomea* (Fig. 3). However, weakly deleterious mutations may still play an additional role in faster-X, as a higher incidence of positive selection on the *X* chromosome than on autosomes is expected to reduce *N*_*eX*_ due to linkage effects [80–82] and result in an associated increased fixation of weakly deleterious mutations.

**Table 2:** Parameters of multiple-regression models and partial correlations for gene expression polymorphism.

	*R* (*P*)	b*Lev (*P*)	b*Spe (*P*)	b*Int (*P*)
*X*	0.38 (<1×10^-12^)	-0.37 (<1×10^-12^)	0.042 (0.094)	-0.001 (0.97)
*A*	0.40 (<1×10^-12^)	-0.32 (<1×10^-12^)	0.16 (<1×10^-12^)	-0.02 (0.1)

*Note*: *R*, correlation coefficient; *P*, Probability; Lev, average gene expression levels; Spe, tissue specificity in gene expression estimated following Yanai *et al*. (2005) [76]; Int, protein interactions identified from Hansen and Kulathinal (2012) [58]; *X*, X-linked genes; *A*, autosomal genes.

### Faster-X evolution of protein-coding sequences in male-biased genes

**Fig 4:**
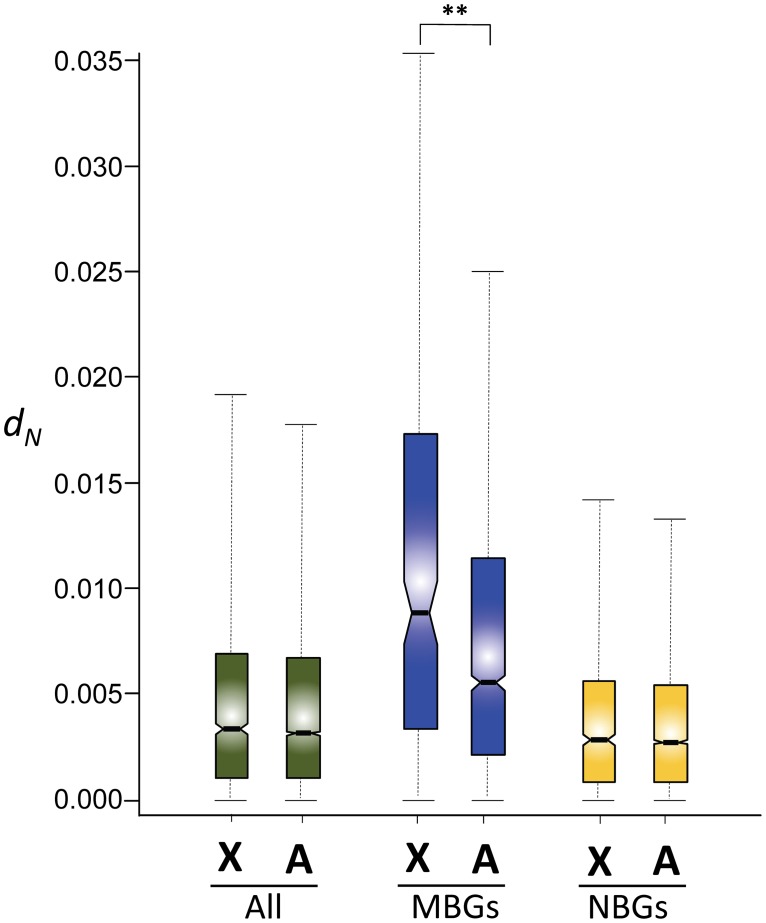
Number of nonsynonymous changes per site (*d*_*N*_) between *D. yakuba* and *D. santomea* in 9,203 genes (green), male-biased genes (blue, MBGs) and nonsex-biased genes (yellow, NBGs). *X*, X-linked genes; *A*, autosomal genes. Asterisks indicate statistically significant differences in *d*_*N*_ (Mann-Whitney *U* test, *P* = 1×10^-6^). (See Fig. 1 legend for explanation of box plots.).

I obtained a high-coverage, high-quality sequence of the *D. santomea* genome that covers >97% of the coding sites annotated in *D. yakuba* (see Materials and Methods for details). To determine whether there is faster-X evolution of protein-coding sequences in *D. yakuba* and *D. santomea*, I examined *d*_*N*_ and *d*_*S*_ in 9,203 genes with information on expression properties and protein interactions (1,480 X-linked and 7,723 autosomal) [43,77]. When all genes are analyzed together, there is no evidence for faster-X in comparisons of *d*_*N*_ (median *d*_*N*_ = 0.003 for X-linked genes and 0.003 for autosomal genes; Mann-Whitney *U* test, *P* = 0.35; Fig. 4) or *d*_*N*_/*d*_*S*_ (*ω*) (median *ω* = 0.074 for X-linked genes and 0.070 for autosomal genes; Mann-Whitney *U* test, *P* = 0.63). To confirm that these trends are representative of whole genome patterns, I also analyzed a larger set of 13,659 genes sequenced in both species (2,335 X-linked and 11,424 autosomal; see Materials and Methods for details), which produced the same non-significant results for *d*_*N*_ (median *d*_*N*_ = 0.0039 for X-linked genes and 0.0038 for autosomal genes; Mann-Whitney *U* test, *P* = 0.76) and marginally significant results for *ω* (median *ω* = 0.092 for X-linked genes and 0.086 for autosomal genes; Mann-Whitney *U* test, *P* = 0.026). The only consistent difference between X-linked and autosomal genes is detected in *d*_*S*_, which is significantly smaller on the *X* chromosome in both gene sets (9,203 genes, median *d*_*S*_ = 0.043 for X-linked genes and 0.045 for autosomal genes; Mann-Whitney *U* test, *P* = 1.3×10^-4^; 13,659 genes, median *d*_*S*_ = 0.041 for X-linked genes and 0.043 for autosomal genes; Mann-Whitney *U* test, *P* < 2.8×10^-8^). This observation is consistent with previous findings of stronger codon bias on the *X* chromosome in other *Drosophila* species, reflecting higher intensity of natural selection for preferred synonymous variants [13,25,28,83–86].

Faster-X evolution is expected to be strongest in MBGs. Tests to the faster-X hypothesis are thus optimally performed when analyzing MBGs and NBGs separately. Two trends became apparent from this analysis. First, MBGs show strong evidence for faster-X evolution at nonsynonymous sites (1,799 genes; median *d*_*N*_ = 0.009 for X-linked genes and 0.006 for autosomal genes; Mann-Whitney *U* test, *P* = 1×10^-6^) but NBGs do not (5,684 genes; median *d*_*N*_ = 0.003 for X-linked genes and 0.003 for autosomal genes; Mann-Whitney *U* test, *P* = 0.5; Fig. 4). Similar patterns are detected for *ω* (*ω* *=* 0.22 *vs*. *ω* = 0.11; Mann-Whitney *U* test, *P* = 2.5×10^-8^ for MBGs and *ω* *=* 0.07 *vs*. *ω* = 0.06; Mann-Whitney *U* test, *P* = 0.053 for NBGs). Second, MBGs appear to evolve significantly faster than NBGs across the genome (median *d*_*N*_ = 0.006 for MBGs and 0.003 for NBGs; Mann-Whitney *U* test, *P* < 1×10^-32^), on the *X* chromosome (median *d*_*N*_ = 0.009 for MBGs and 0.003 for NBGs; Mann-Whitney *U* test, *P* = 2.6×10^-28^) and on autosomes (median *d*_*N*_ = 0.006 for MBGs and 0.003 for NBGs; Mann-Whitney *U* test, *P* < 1×10^-32^). All together these results support the idea that MBGs and NBGs evolve under different selective regimes, possibly connected with differences in function [46,87–92], pleiotropic effects, and/or differences in expression properties [46,47] and protein interactions [65].

Genomewide correlations of gene expression levels, tissue specificity, and the number of protein interactions with *d*_*N*_, *d*_*S*_ and *ω*, open the possibility that faster-X could be the by-product of differences in these properties between X-linked and autosomal genes (Table 3). To take into account this possibility, I applied a multiple-regression analysis (Table 4). Residuals of *d*_*N*_ are significantly greater for X-linked than for autosomal MBGs (Mann-Whitney *U* test, *P* = 6×10^-5^) but this difference is not observed in NBGs (Mann-Whitney *U* test, *P* = 0.48 for NBGs). In addition, the comparison of divergence between genes with similar expression properties and number of protein interactions using a nonparametric test indicates that MBGs on the *X* chromosome show consistently higher *d*_*N*_ than autosomal MBGs (Wilcoxon matched pairs test, *T* = 4615.5, *P* = 0.014; see above). Both results imply that faster-X protein evolution in MBGs of *D. yakuba* and *D. santomea* remains detectable even after correcting for differences between the *X* chromosome and autosomes in expression levels, tissue specificity, and number of protein interactions.

**Table 3:** Spearman’s rank correlations (*ρ*) of *d*_*N*_, *d*_*S*_ and *ω* with gene expression levels, tissue specificity, and protein interactions.

		*ρ* (*P*)	
	*d*_*N*_	*d*_*S*_	*ω*
**Levels**			
MBGs	-0.28 (<0.001)	-0.094 (<0.001)	-0.23 (<0.001)
NBGs	-0.23 (<0.001)	-0.13 (<0.001)	-0.17 (<0.001)
All	-0.24 (<0.001)	-0.12 (<0.001)	-0.19 (<0.001)
**Specificity**			
MBGs	0.30 (<0.001)	0.10 (<0.001)	0.24 (<0.001)
NBGs	0.22 (<0.001)	0.02 (0.13)	0.21 (<0.001)
All	0.33 (<0.001)	0.057 (<0.001)	0.30 (<0.001)
**Interactions**			
MBGs-	-0.24 (<0.001)	-0.045 (0.06)	-0.21 (<0.001)
NBGs	-0.23 (<0.001)	-0.07 (<0.001)	-0.19 (<0.001)
All	-0.31 (<0.001)	-0.07 (<0.001)	-0.27 (<0.001)

*Note*: *P*, Probability; MBGs, 1,799 male-biased genes; NBGs, 5,684 nonsex-biased genes; All, 9,203 genes.

**Table 4:** Parameters of multiple-regression models and partial correlations for rates of protein-coding sequence evolution.

	*R* (*P*)	b*Lev (*P*)	b*Spe (*P*)	b*Int (*P*)
*ω*				
MBG	0.21 (<1×10^-12^)	-0.11 (<5×10^-6^)	0.14 (<7×10^-7^)	-0.03 (ns)
NBG	0.17 (<1×10^-12^)	-0.07 (<1×10^-12^)	0.13 (<1×10^-12^)	-0.002 (ns)
All	0.26 (<1×10^-12^)	-0.06 (<3.7×10^-9^)	0.23 (<1×10^-12^)	-0.009 (ns)
***d***_***N***_				
MBG	0.30 (<1×10^-12^)	-0.16 (<1×10^-12^)	0.21 (<1×10^-12^)	-0.03 (ns)
NBG	0.26 (<1×10^-12^)	-0.13 (<1×10^-12^)	0.18 (<1×10^-12^)	-0.03 (0.012)
All	0.36 (<1×10^-12^)	-0.10 (<1×10^-12^)	0.31 (<1×10^-12^)	-0.029 (0.0027)
***d***_***S***_				
MBG	0.13 (<1×10^-12^)	-0.05 (0.03)	0.10 (<9×10^-5^)	0.001 (ns)
NBG	0.12 (<1×10^-12^)	-0.12 (<1×10^-12^)	-0.04 (0.032)	-0.03 (0.011)
All	0.11 (0.026)	-0.10 (<1×10^-12^)	0.02 (ns)	-0.03 (0.008)

*Note*: *R*, correlation coefficient; *P*, Probability; Lev, average gene expression levels; Spe, tissue specificity in gene expression estimated following Yanai *et al*. (2005) [76]; Int, protein interactions identified from Hansen and Kulathinal (2012) [58]; MBG, male-biased genes; NBGs, nonsex-biased genes; All, all genes; ns, *P* > 0.05.

Although expression levels, tissue specificity, and the number of protein interactions are important to our understanding of X-linked divergence of both gene expression and protein sequences in the *D. yakuba*—*D. santomea* system, additional factors are necessary to explain faster-X evolution. The hemizygosity of the *X* chromosome in males has been often used as the default explanation [1,2]. Faster-male evolution driven by positive selection [93,94] could additionally facilitate faster-X for MBGs in systems where males are the heterogametic sex [95]. Other differences between the *X* chromosome and autosomes such as recombination rates could also be contributing factors to faster-X [19]. Because *Drosophila* males lack crossing over, the *X* chromosome is more exposed to recombination than the autosomes, as it spends 2/3 of its time in females [9,11,19]. The difference is particularly exaggerated in *D. melanogaster*, in which the *X* chromosome also shows an increased rate of recombination per female meiosis relative to that of autosomes [96] and there are abundant autosomal polymorphic inversions [31]. As a result of higher recombination, the efficacy of selection is expected to be higher on the *X* chromosome than on autosomes, which could potentially lead to a higher incidence of adaptive evolution on the *X* chromosome [13,21].

## Conclusions

I report here parallel trends of faster-X evolution of gene expression and protein-coding sequences in *D. yakuba* and *D. santomea*. The increased divergence is specific to X-linked MBGs and not detected in NBGs. Multiple-regression and nonparametric analyses indicate that faster-X is not the by-product of differences between X-linked and autosomal genes in properties known to correlate with rates of evolution, such as gene expression levels, tissue specificity in expression, and the number of protein interactions. Because MBGs are significantly less polymorphic for gene expression than NBGs on the *X* chromosome, the observed excess of gene expression divergence is consistent with a higher incidence of positive selection, possibly associated with the hemizygosity of the *X* chromosome in males. Besides male heterogamety, other differences between X-linked and autosomal genes, such as recombination rates, could also contribute to faster-X. Note that an additional contribution of weakly deleterious mutations to faster-X cannot be ruled out, as recurrent adaptive evolution on the *X* chromosome will inevitably reduce *N*_*eX*_ due to linkage effects, which in time will result in an increased fixation of weakly deleterious mutations. It is thus possible that the excess of X-linked divergence is the compound effect of both beneficial and weakly deleterious mutations.

## Supporting Information

S1 Fig Gene expression divergence between *D. yakuba* and *D. santomea*. Gene expression divergence (*D*_*e*_) is shown for all genes analyzed (green, All), male-biased genes (blue, MBGs), and nonsex-biased genes (yellow, NBGs). *X*, X-linked genes; *A*, autosomal genes. The heavy horizontal line in each box indicates the median. The length of the box and the whiskers represent 50% and 90% confidence intervals, respectively. Asterisks indicate significant differences (Mann-Whitney *U* test; **, *P* < 0.001).
(PDF)
