# Supplementary figures and images for: Parallel Faster-X Evolution of Gene Expression and Protein Sequences in Drosophila: Beyond Differences in Expression Properties and Protein Interactions
Source: PLoS One. 2015 Mar 19;10(3):e0116829. doi: 10.1371/journal.pone.0116829 (PMC4366066; doi:10.1371/journal.pone.0116829)

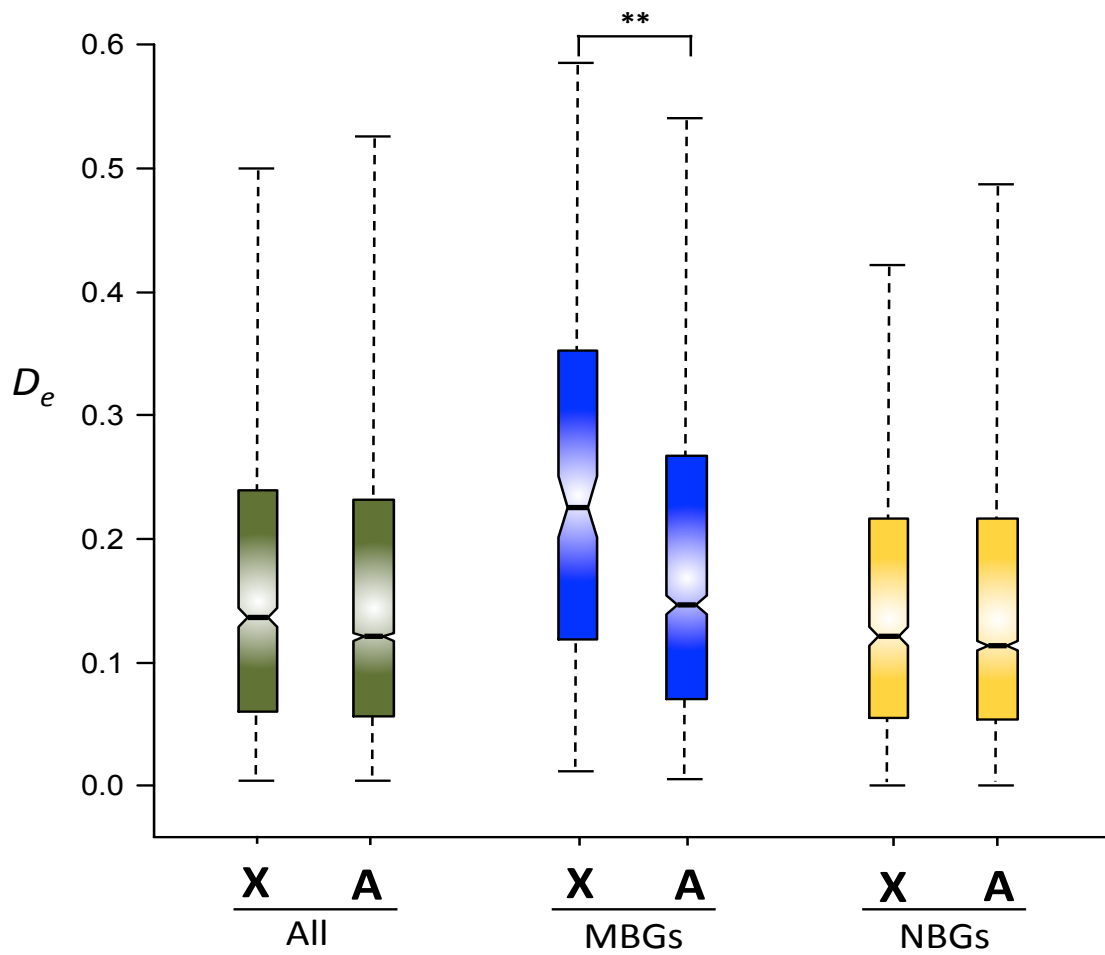

Supplement: S1 Fig — Gene expression divergence (D e) is shown for all genes analyzed (green, All), male-biased genes (blue, MBGs), and nonsex-biased genes (yellow, NBGs). X, X-linked genes; A, autosomal genes. The heavy horizontal line in each box indicates the median. The length of the box and the whiskers represent 50% and 90% confidence intervals, respectively. Asterisks indicate significant differences (Mann-Whitney U test; **, P < 0.001). (PDF) [file pone.0116829.s001.pdf]
